# Efficacy of a New Multivalent Vaccine for the Control of Bovine Respiratory Disease (BRD) in a Randomized Clinical Trial in Commercial Fattening Units

**DOI:** 10.3390/vaccines12111233

**Published:** 2024-10-29

**Authors:** Mariona Tapiolas, Marta Gibert, Carlos Montbrau, Ester Taberner, Marina Solé, Héctor Santo Tomás, Ainhoa Puig, Ricard March

**Affiliations:** 1 Hipra Scientific S.L.U., Avda. La Selva 135, 17170 Amer, Spain; marta.gibert@hipra.com (M.G.); carlos.montbrau@hipra.com (C.M.); ester.taberner@hipra.com (E.T.); ainhoa.puig@hipra.com (A.P.); ricard.march@hipra.com (R.M.); 2 HIPRA S.A., Avinguda La Selva 135, 1710 Amer, Spain; marina.sole@hipra.com (M.S.); hector.santotomas@hipra.com (H.S.T.)

**Keywords:** bovine respiratory disease (BRD), morbidity, incidence, antimicrobials, field conditions, performance, vaccine

## Abstract

A new multivalent vaccine (DIVENCE^®^), containing live gE/tk double-gene-deleted BoHV-1, live-attenuated BRSV, inactivated PI3, and BVDV-1, and BVDV-2 recombinant proteins, has been designed to protect cattle against the main viral pathogens associated with bovine respiratory disease (BRD). The aim of this study was to demonstrate the efficacy of DIVENCE^®^ against BRD in field conditions. A total of 360 animals from three different farms were included in this study. Calves were randomly distributed to the vaccinated (*n* = 183; DIVENCE^®^) or control (*n* = 177; phosphate-buffered saline solution) group. All animals received two intramuscular doses (2 mL/dose) three weeks apart of the corresponding product. The entire fattening period (approximately 9 months) was monitored to assess the incidence, severity, and morbidity of BRD as well as administered treatments and growth performance. During this study, a BRSV outbreak was reported in one farm, where vaccinated animals had significantly (*p* < 0.02) lower morbidity (20.4%) and severity (score of 1.70) compared to the control group (53.70% and score of 2.11). Overall, vaccinated animals had a significantly lower number of cases (*p* < 0.001; 0.36 vs. 0.64 cases/calf), lower morbidity (*p* < 0.004; 26.78% vs. 41.24%), and lower antimicrobial treatments (*p* = 0.01; 33.3% vs. 57.4%) than control animals. Vaccinated animals presented significantly (*p* = 0.01) higher carcass weight than controls (6.58 kg). Vaccination with DIVENCE^®^ at the beginning of the fattening period decreased the incidence and morbidity of BRD following a BRSV outbreak. Additionally, the overall incidence and morbidity of BRD throughout the entire fattening period were reduced across farms. Thus, DIVENCE^®^ can improve economic outcomes in fattening units by reducing antibiotic treatments and enhancing performance.

## 1. Introduction

Bovine respiratory disease (BRD) is the most common cause of morbidity and mortality in cattle [1]. This disease is a multifactorial syndrome and is mostly associated with bovine respiratory syncytial virus (BRSV), infectious bovine rhinotracheitis (IBR), bovine viral diarrhea virus (BVDV), and bovine parainfluenza virus 3 (PI-3). All these viruses are known to be involved alone or in combination with each other and/or with bacteria in the pathogenesis of BRD [2]. BRD is often the result of interactions between stress and bacterial and viral infections. In feedlots, BRD is usually observed in the first period of the fattening phase when animals must cope with stressful events such as transportation, commingling, and weaning [3,4]. The negative economic impact of BRD due to the increase in mortality and treatment costs (metaphylactic and therapeutic use of antimicrobials) alongside the reduction in performance and carcass value has been reported by several authors worldwide [2,5,6,7,8]. Furthermore, a high incidence of BRD increases the use of antibiotics, which spurs an increase in antibiotic resistance [9]. These effects, coupled with the growing concern for animal welfare, create a need to find solutions to improve animal health through other strategies, such as prevention. The effects of BRD are more marked during the first weeks after arrival in calf-rearing units when mortality and morbidity rates are usually high [10,11]. Controlling the BRD complex involves improving both environmental and management conditions while preventing viral and bacterial infections through vaccination [12,13,14]. One common management practice is to vaccinate young calves early in life to provide immune protection before stressful situations, although vaccination is usually performed upon arrival on the feedlot [15]. The objective of these measures is to improve the feedlot health program in order to minimize the incidence and costs associated with morbidity and mortality (BRD and other diseases) through designated prevention and control programs, thus maximizing feeding performance and carcass value [16]. Recently, a new multivalent vaccine (DIVENCE^®^), containing live gE-/tk- double-gene-deleted BoHV1, live-attenuated BRSV, and inactivated PI3 and BVDV-1 recombinant protein and BVDV2 recombinant protein, has been designed to protect cattle against the main viral pathogens associated with BRD. The efficacy of the vaccine against these viruses has been demonstrated in experimental conditions, with protection against BRD being achieved. The vaccine is also effective for fetal protection in animals challenged with ncp BVDV-1 and BVDV-2 [17]. The aim of this randomized field trial was to evaluate the efficacy of the DIVENCE^®^ vaccine in young calves in reducing the incidence, morbidity, and need for treatment for BRD while enhancing average daily weight gain (ADWG) on three different commercial fattening units.

## 2. Materials and Methods

The protocol of this study was reviewed and approved by the Spanish Agency for Medicine and Medical Devices (20/008 ECV (20200716/V/24/001)) and conducted in accordance with the guidelines on Good Clinical Practices [18].

### 2.1. Study Animals (Animals and Housing)

This study was conducted on three commercial cattle feedlots located in the northeast region of Spain. On each feedlot, a single batch of animals was included in this study. A batch was considered as the group of calves that arrived at the farm on the same day and remained together for the entire fattening period. All animals enrolled in this study were approx. ten weeks of age (73.4 ± 0.6 days of age), with the minimum age at vaccination being 55 days at Day 0 of this study. No animals had been previously vaccinated against BRSV, BoHV1 (IBR), PI3, BVDV1, or BVDV2. A total of 360 animals from three different batches were included in this study: one batch of 108 Holstein–Friesian males (Farm 1), another batch of 99 Holstein–Friesian males (Farm 2), and another batch of 153 Belgian-Blue cross males and females (Farm 3). Each batch remained housed in the same airspace for the entire fattening period in outdoor pens that were open at the front and partially closed at the back. All animals were commingled together throughout this study. All animals were fed ad libitum using concentrate and forage to meet nutritional requirements.

### 2.2. Study Design

This study was a controlled, randomized, double-blind, parallel field trial. On the vaccination day (Day 0 of this study; D0), calves from the same batch were randomly distributed between the two study groups. The vaccinated group (n = 183) received the DIVENCE^®^ vaccine and the control group (n = 177) received a placebo injection of phosphate-buffered saline (PBS) solution. Both groups were given two intramuscular doses (2 mL/dose) of the corresponding product three weeks apart. The personnel involved in the vaccination procedure conducted no other study-related tasks.

### 2.3. Clinical Observations

Animals were checked daily by the farmer and weekly by the veterinarian from vaccination (D0) until the end of the fattening period, approx. 9 months after vaccination. During this period, animals showing respiratory clinical signs were considered as a ‘case of respiratory disease (RD)’. A score was given to all RD cases using a 4-point scale to evaluate the severity of clinical signs based on previous work [19]. This score was as follows: 0: clinically normal; (1): mild signs of depression with mild respiratory signs; (2): moderate signs of depression with moderate respiratory distress; (3): severe signs of depression with severe respiratory distress; and 4: moribund.

An outbreak was considered if more than 10% of animals showed signs of RD for 2–3 consecutive days following the recommendations described by the Spanish Ministry of Agriculture, Fisheries, and Food [20]. Animals were then monitored daily for five days after the outbreak. Nasal exudate samples from the reported cases were also collected to detect specific pathogens by means of PCR.

Antimicrobial metaphylactic treatments were not allowed during the entire study period so that RD could be properly explored. If a calf presented respiratory signs, individual antimicrobial treatment was administered per criteria from the farmer/veterinarian. Individual treatments with respiratory purposes and cases of mortality were recorded during the overall follow-up period. Necropsies were performed by the veterinarian whenever possible.

### 2.4. Performance Evaluation

Days on feed were calculated from D0 to slaughter (342 ± 32.1 days of age). Individual live body weight was recorded at D0, and individual carcass weight was obtained from the slaughterhouse. Average daily weight gain (ADWG, g/day) was calculated as the difference between live body weight at D0 and the estimated final body weight in relation to days on feed. The estimated final live body weight was determined considering a 55% dressing percentage [21].

### 2.5. Data Analysis

Animals that did not complete the vaccination schedule (2 doses, 21 days apart) were excluded from the statistical analysis. The statistical analysis was performed using R software v3.1. A *p*-value of <0.05 was considered as the limit for statistical significance. To compare the two groups, four analyses were performed per variable: one for each feedlot and one overall. In the overall analysis, the farm was used as a random effect. The overall incidence of RD was analyzed using a Poisson regression model and a Cox proportional hazards survival model. Morbidity was analyzed using a logistic regression model, as well as odds ratio and a Cox survival model. The number of treatments (one or more antimicrobial treatments) and mortality were analyzed using a logistic regression model. A Cox survival model was also performed for mortality. Productivity data (days on feed, weight at Day 0, growth, and carcass weight) were analyzed using a *t*-test on each farm or a linear regression model (overall test).

## 3. Results

### 3.1. Incidence, Mortality, and Antimicrobial Treatments Due to Respiratory Disease

A total of 360 calves were followed up until the end of fattening. At the beginning of this study (D0), no statistical differences were observed between groups in terms of age and body weight, as detailed in Table 1.

During the study period, the percentage of vaccinated animals on Farms 1 and 2 that presented at least one episode of RD was significantly (*p* < 0.05) lower compared to morbidity in the control groups (Table 2). Low morbidity was observed in both groups on Farm 3. Overall, vaccinated animals had significantly (*p* < 0.004) lower morbidity than controls, with only 49 out of the 183 vaccinated calves (26.78%) presenting at least one episode of RD versus 73 out of the 177 control calves (41.24%). Thus, a reduction of 35.1% in morbidity was observed in vaccinated animals.

Regarding the total number of RD cases reported during the entire study, vaccinated animals on Farm 1 were observed to have significantly (*p* < 0.05) fewer cases than controls. On Farms 2 and 3, the number of cases in vaccinated groups was numerically lower than in control groups. Overall, vaccinated animals had significantly (*p* < 0.001) fewer cases than control animals, with only 66 cases reported in the 183 vaccinated calves (0.36 cases/calf) versus 110 cases in the 177 control calves (0.62 cases/calf). A reduction of 42% in cases was observed in the vaccinated animals.

During the study follow up, one respiratory outbreak was reported in one of the farms (Farm 1) on Day 23, just two days after the second dose. Laboratory results confirmed BRSV as the only etiological pathogen responsible for the outbreak. On this farm, during the outbreak follow up, the vaccinated group had significantly (*p* < 0.001) fewer animals affected by RD (11 out of 54; 20.4%) than the control group (29 out of 54; 53.70%). Consequently, a reduction in RD cases of 62.1% in the vaccinated group was observed. The odds of having RD were 0.22 (95% CI: 0.22 [0.090.52]) in vaccinated animals versus control animals; control animals were thus 4.5 times more likely to suffer from RD. The severity score of clinical signs during the outbreak was also reported to be significantly (*p* < 0.02) lower in the vaccinated group (1.70) than the control group (2.11). During the outbreak, five calves died due to RD. Findings at necropsy showed acute pulmonary emphysema with extensive pulmonary lesions, consistent with BRSV. The mortality rate reported in the vaccinated group was numerically lower than in the control group (1.85% versus 7.41%).

In terms of the antimicrobial treatments reported for RD during the study follow up, the percentage of animals that required at least one treatment in the vaccinated group on Farm 1 was significantly (*p* = 0.01) lower than the control group (33.3% vs. 57.4%). On Farm 2, the percentage of calves that required at least one treatment was also lower in the vaccinated group in comparison to the control group (27.4% vs. 43.7%). On Farm 3, the groups were found to be similar in this regard (22.7% vs. 21.8%). Overall, vaccinated animals needed significantly (*p* = 0.01) fewer antimicrobial treatments (at least one due to RD) than controls. Similar results were observed for the total number of calves (177 vs. 183) that required more than one antimicrobial treatment for RD (29.6% vs. 13.0%, 12.5% vs. 3.9%, and 5.3% vs. 5.13% for Farms 1, 2, and 3 respectively). Overall, the percentage of vaccinated animals that received more than one antimicrobial treatment (14.7% vs. 7.1%) was significantly (*p* < 0.02) lower than in the control group.

### 3.2. Productivity Parameters

Animals spent an average of 269 days in this study from vaccination until slaughter. The groups were considered homogeneous in terms of initial body weight (*p* = 0.34). The performance results are summarized in Table 3. ADWG and carcass weight were found to be greater in vaccinated animals compared to controls on all three farms. Overall, vaccinated animals presented numerically higher ADWG (*p* = 0.07) and significantly higher carcass weight (*p* = 0.01) than controls at 35.78 g/day and 6.58 kg, respectively.

When ADWG and carcass weight were compared between animals with RD and those without, higher ADWG was still observed in vaccinated animals despite RD. Comparable results were observed for carcass weight, with overall higher results (*p* = 0.08) for carcass weight in vaccinated animals with RD than corresponding controls.

## 4. Discussion

The objective of the present study was to evaluate the efficacy of a pentavalent viral vaccine (DIVENCE^®^) under field conditions, focusing on the assessment of incidence, morbidity, and antimicrobial treatments related to BRD and productivity parameters in different feedlots. During this study, one BRD outbreak was observed on Farm 1. This outbreak occurred 23 days after the start of the vaccination program, two days after the second dose (98.7 ± 8.67 days of age). This rapid onset of a BRD outbreak during the first days of fattening is a common occurrence [16,22]; in a previous study [23] involving over 12,000 fattening calves, it was found that 80% of cases of BRD were observed within the first 55 days after entering the feedlot. Despite the outbreak occurring immediately after completion of the vaccination program, a significant (*p* < 0.01) reduction in incidence was observed in vaccinated animals compared to controls. Laboratory results identified BRSV as the etiological pathogen; lung lesions were also compatible with this. BRSV can be considered one of the main pathogens in BRD [24,25], especially during the first year of life [26,27]. The results obtained demonstrate that the attenuated vaccine virus strain of BRSV contained in DIVENCE^®^ induces a protective immunological response. This vaccine strain has been proven efficacious against an experimental BRSV infection when used intranasally in young animals [26] several weeks after vaccination. The results observed in the present study suggest that the vaccine induces both a humoral and cellular response, resulting in a rapid response of vaccinated animals to the natural infection. This cellular response was previously described using a vaccine containing the same attenuated BRSV strain [26]. The most relevant clinical signs observed during the outbreak were dyspnea followed by nasal discharge and cough; few animals died. The significant differences in clinical signs observed were between vaccinated and control animals, suggesting that the vaccine reduced the severity of the infection.

During this study, the vast majority of RD cases were reported during the first months of the follow-up period. Specifically, 52% of RD cases were reported within the first month after initiation of this study and 43% between the first and third month after day D0. Only 6% of RD cases were observed afterwards. These results confirm that most BRD cases occur during the first days of the fattening period, as other authors have described [16,22,23]. These findings reveal the importance of prevention and control of respiratory disease during the first months of fattening. Farm 1 suffered the highest incidence and morbidity of RD cases during the study period because of the respiratory outbreak. Nevertheless, the incidence of RD on Farm 2, where no outbreak was reported, was significantly lower in the vaccinated group (27.4%) compared to the control group (47.9%). Farm 3 exhibited a similar incidence and morbidity across both groups, with the lowest incidence in the control group among the enrolled feedlots. These differences in incidence and morbidity between farms could be related to the different characteristics of the farms, where different breeds, facilities, management, stocking densities, and weather conditions are noticed between them. Furthermore, the occurrence of an outbreak is unpredictable, and some farms could have been more prone to the occurrence of it. For example, animals from Farm 3 came from nearby farms, whereas animals from Farms 1 and 2 were transported from other countries, suffering from transport stress. However, considering all data from all three feedlots, the overall incidence and morbidity of RD cases was significantly lower in vaccinated animals, which suggests that the effect of viruses associated with BRD had a lesser impact on those animals.

This decrease in incidence, severity, and morbidity may favor a reduction in antibiotic treatment. It is well known that mixed infections are frequently identified in BRD, and bacteria are BRD-associated pathogens [28]. It also is likely that prior viral infections (BRSV, PI3, IBR, or BVDV) can predispose cattle to severe bacterial bronchopneumonia while going undiagnosed due to the overwhelming bacterial lesions [29]. Antibiotics, however, target bacteria and mycoplasma and are not effective against viruses. Some authors [30] have described BRSV infections actually occurring after an animal is treated with antibiotics for a bacterial infection, which raises concerns about massive antibiotic use and consequent antibiotic resistance. On the other hand, if BRD is not properly diagnosed, its delayed detection can impair the efficacy of the treatment and the expected recovery [31]. In many cases, antimicrobial treatment begins when clinical signs are already very evident, so it is very likely that lung damage has already occurred. This can be detrimental to short- and long-term health status because lung consolidation is linked to increased mortality, delayed growth, lower reproductive performance, and shorter life expectancy [32]. Prevention is key to improving animal welfare, complying with new legislation that calls for a reduction in the use of antibiotics [33] (i.e., Regulation (EU) 2019/6 in the European Union) and above all minimizing the costs associated with BRD. In the present study, a clear and significant reduction in antibiotic treatment in the overall analysis was observed. This reduction could be explained by vaccine-induced protection against the most relevant BRD viruses. Viral agents, such as BRSV, BHV-1, BVDV, parainfluenza-3 virus, and others, further weaken the immune defenses of calves, paving the way for bacterial infections that lead to serious respiratory issues [34,35,36].

The differences observed between vaccinated and control animals had an impact on performance. Overall, higher ADWG (g/d) and carcass weight were observed in vaccinated animals compared to control animals. This result was observed when considering all the animals, only those animals with RD, and only those animals without RD. The negative impact of BRD on performance has already been widely reported. The consequences of BRD in young calves include reduced ADWG [37,38]. In animals with BRD during their first month of fattening, ADWG can fall by 370 g/day [4]. While there may be compensatory growth afterwards, their total growth compared to the healthy counterparts in the group will not recover. Thus, ADWG in animals with BRD is reduced by 70–90 g/day overall compared to healthy calves [4,39]. This reduction in ADWG impacts days on feed and/or carcass weight. In addition to the cost of treating animals with BRD, there is another cost that can be overlooked: the extra days spent finishing by animals that have had BRD [8]. This can amount to an additional 5.5 days until slaughter for every four and a half months on feed [5]. In the present study, animals in both groups (vaccinated and control) had similar body weights at the beginning of the fattening period and had practically the same number of days on feed. However, the overall average carcass weight was significantly higher in the vaccinated group compared to control animals. This indicates that the performance of vaccinated animals was greater, suggesting that the reduction in BRD induced by the vaccine minimizes the impact on the carcass. It should also be noted that subclinical BRD, without mortality or even associated veterinary costs, can still induce a substantial reduction in ADWG and carcass quality [40,41]. It is estimated that, at best, only 50% of animals with BRD show respiratory symptoms [42]. On Farm 3, lower incidence and morbidity values were observed compared to the other farms, but vaccinated animals had significantly higher carcass weights compared to controls, suggesting that undetected or subclinical BRD cases impair performance by impacting carcass weight in the long term. Consequently, the use of the vaccine reduced the incidence and morbidity of the most relevant BRD viruses, preventing bacteria from easily colonizing the respiratory tissue and thereby reducing the number of antibiotic treatments. In summary, by minimizing the impact of BRD on the herd through vaccination, i.e., decreasing the incidence and morbidity of BRD as well as antibiotic treatments, higher performance in terms of carcass weight was achieved.

## 5. Conclusions

The overall incidence and morbidity of BRD were reduced in vaccinated animals throughout the fattening period among the study. Additionally, vaccination with DIVENCE^®^ at the beginning of the fattening period decreased the incidence and morbidity of BRD following a BRSV outbreak only two days after the primary vaccination scheme. Thus, DIVENCE^®^ can improve the economic outcomes in fattening units by reducing antibiotic treatments and enhancing performance.

## 6. Patents

A patent application has been filed by HIPRA SCIENTIFIC S.L.U. on vaccines against bovine viral diarrhea virus and bovine respiratory disease, including the novel vaccine DIVENCE. M. Gibert is one of the inventors of said patent application.

## Figures and Tables

**Table 1:** Descriptive statistics of study animals on vaccination day (D0).

	Farm 1		Farm 2		Farm 3	
	Control	Vacc.	Control	Vacc.	Control	Vacc.
Animals (N)	54	54	48	51	75	78
Mean age (days) ± SE	75.2 ± 1.18	76.4 ± 1.20	76.0 ± 1.67	74.4 ± 1.39	69.3 ± 1.29	71.7 ± 1.78
*p*-value	0.34		0.67		0.76	
Mean weight (kg) ± SE	92.0 ± 1.76	91.1 ± 1.77	97.1 ± 1.47	95.3 ± 1.35	100.5 ± 2.21	105.2 ± 2.32
*p*-value	0.74		0.37		0.14	
	Overall					
	Control	Vacc.				
Animals (N)	177	183				
Mean age (days) ± SE	72.9 ± 0.82	73.8 ± 0.93				
*p*-value	0.68					
Mean weight (kg) ± SE	97.0 ± 1.18	98.3 ± 1.26				
*p*-value	0.34					

**Table 2:** Prevalence of RD for each individual feedlot and overall data during the follow-up period.

	Farm 1		Farm 2		Farm 3		Overall		
	Control	Vacc.	Control	Vacc.	Control	Vacc.	Control		Vacc.
At least one case of RD									
Number of calves	31	18	23	14	19	17	73		49
Percentage (%)	57.4	33.3	47.9	27.4	25.3	21.8	41.2		26.8
*p*-value	0.01		0.04		0.61		0.004		
Odds ratio	0.37 (0.17–0.81)		0.45 (0.18–0.95)		0.82 (0.39–1.74)		0.52 (0.33–0.81)		
Total cases of RD									
Number of cases	58	24	27	17	25	25	110		66
Cases/calf	1.07	0.44	0.56	0.33	0.33	0.32	0.62		0.36
*p*-value	<0.001		0.09		0.89		<0.001		

**Table 3:** Performance results for each individual feedlot and overall data during the follow-up period (mean ± SE).

	Farm 1		Farm 2		Farm 3		Overall	
	Control	Vacc.	Control	Vacc.	Control	Vacc.	Control	Vacc.
Days on feed (d)	303.7 ± 1.16	301.9 ± 1.86	265.5 ± 2.45	266.5 ± 3.36	247.0 ± 2.32	248.4 ± 2.17	268.5 ± 2.21	269.1 ± 2.19
*p*-value	0.41		0.82		0.67		0.85	
ADWG (g/d)								
Overall	1313.5 ± 20.02	1345.6 ± 17.17	1364.7 ± 15.26	1391.3 ± 19.25	1357.5 ± 22.97	1402.4 ± 22.71	1346.8 ± 12.32	1382.6 ± 12.28
*p*-value	0.23		0.28		0.17		0.07	
RD	1286 ± 30.29	1346 ± 32.41	1343 ± 20.73	1344 ± 40.75	1268 ± 45.91	1329 ± 41.57	1300 ± 18.75	1340 ± 21.56
*p*-value	0.18		0.98		0.33		0.17	
No RD	1345 ± 24.46	1345 ± 20.44	1386 ± 22.18	1408 ± 21.38	1397 ± 25.07	1418 ± 26.07	1383 ± 15.71	1396 ± 14.56
*p*-value	0.99		0.47		0.55		0.46	
Carcass weight (kg)								
Overall	269.6 ± 3.59	273.7 ± 3.07	252.2 ± 2.26	256.0 ± 3.26	238.2 ± 2.86	247.8 ± 2.47	251.1 ± 1.99	257.7 ± 1.84
*p*-value	0.39		0.33		0.012		0.01	
RD	263 ± 4.81	275 ± 5.54	249 ± 3.26	248 ± 4.49	225 ± 5.88	236 ± 5.77	248 ± 3.26	254 ± 3.95
*p*-value	0.13		0.78		0.17		0.08	
No RD	277 ± 5.09	273 ± 3.74	255 ± 2.71	259 ± 4.04	243 ± 2.97	250 ± 2.61	253 ± 2.43	259 ± 2.06
*p*-value	0.58		0.42		0.079		0.19	

## Data Availability

The data presented in this study are available from the corresponding author upon reasonable request.

## References

[1] Holman D.B., McAllister T.A., Topp E., Wright A.D., Alexander T.W. (2015). The nasopharyngeal microbiota of feedlot cattle that develop bovine respiratory disease. Vet. Microbiol..

[2] Fulton R.W. (2009). Bovine respiratory disease research (1983–2009). Anim. Health Res. Rev..

[3] Timsit E., Bareille N., Seegers H., Lehebel A., Assié S. (2011). Visually undetected fever episodes in newly received beef bulls at a fattening operation: Occurrence, duration, and impact on performance. J. Anim. Sci..

[4] Schneider M.J., Tait R.G., Busby W.D., Reecy J.M. (2009). An evaluation of bovine respiratory disease complex in feedlot cattle: Impact on performance and carcass traits using treatment records and lung lesion scores. J. Anim. Sci..

[5] Thompson P.N., Stone A., Schultheiss W.A. (2006). Use of treatment records and lung lesion scoring to estimate the effect of respiratory disease on growth during early and late finishing periods in south African feedlot cattle. J. Anim. Sci..

[6] Cusack P.M., Mc Meniman N.P., Lean I.J. (2007). Feedlot entry characteristics and climate: Their relationship with cattle growth rate, bovine respiratory disease and mortality. Aust. Vet. J..

[7] Pardon B., Hostens M., Duchateau L., Dewulf J., De Bleecker K., Deprez P. (2013). Impact of respiratory disease, diarrhea, otitis and arthritis on mortality and carcass traits in white veal calves. BMC Vet. Res..

[8] Fernández M., Ferreras M.D.C., Giráldez F.J., Benavides J., Pérez V. (2020). Production Significance of Bovine Respiratory Disease Lesions in Slaughtered Beef Cattle. Animals.

[9] Catry B., Dewulf J., Maes D., Pardon B., Callens B., Vanrobaeys M., Opsomer G., de Kruif A., Haesebrouck F. (2016). Effect of Antimicrobial Consumption and Production Type on Antibacterial Resistance in the Bovine Respiratory and Digestive Tract. PLoS ONE.

[10] Pardon B., De Bleecker K., Hostens M., Callens J., Dewulf J., Deprez P. (2012). Longitudinal study on morbidity and mortality in white veal calves in Belgium. BMC Vet. Res..

[11] Winder C.B., Kelton D.F., Duffield T.F. (2016). Mortality risk factors for calves entering a multi-location white veal farm in Ontario, Canada. J. Dairy Sci..

[12] Pratelli A., Cirone F., Capozza P., Trotta A., Corrente M., Balestrieri A., Buonavoglia C. (2021). Bovine respiratory disease in beef calves supported long transport stress: An epidemiological study and strategies for control and prevention. Res. Vet. Sci..

[13] Fulton R.W., d’Offay J.M., Eberle R., Moeller R.B., Campen H.V., O’Toole D., Chase C., Miller M.M., Sprowls R., Nydam D.V. (2015). Bovine herpesvirus-1: Evaluation of genetic diversity of subtypes derived from field strains of varied clinical syndromes and their relationship to vaccine strains. Vaccine.

[14] Cusack P.M. (2023). Evaluation of practices used to reduce the incidence of bovine respiratory disease in Australian feedlots (to November 2021). Aust. Vet. J..

[15] Jourquin S., Lowie T., Debruyne F., Chantillon L., Clinquart J., Pas M.L., Boone R., Hoflack G., Vertenten G., Sustronck B. (2023). Effect of on-arrival bovine respiratory disease vaccination on ultrasound-confirmed pneumonia and production parameters in male dairy calves: A randomized clinical trial. J. Dairy Sci..

[16] Edwards T.A. (2010). Control methods for bovine respiratory disease for feedlot cattle. Vet. Clin. N. Am. Food Anim. Pract..

[17] Taberner E., Gibert M., Montbrau C., Muñoz I., Mallorquí J., Santo Tomás H., Prenafeta A., March R. (2024). Fetal protection against bovine viral diarrhea virus types 1 and 2 after vaccination of the dam with the DIVENCE vaccine. BioRxiv.

[18] European Medicine Agency, VICH GL9 Good clinical practices–Scientific guideline EMEA/CVMP/VICH/595/98. https://www.ema.europa.eu/en/vich-gl9-good-clinical-practices-scientific-guideline.

[19] Torres S., Thomson D.U., Bello N.M., Nosky B.J., Reinhardt C.D. (2013). Field study of the comparative efficacy of gamithromycin and tulathromycin for the treatment of undifferentiated bovine respiratory disease complex in beef feedlot calves. Am. J. Vet. Res..

[20] Ministry of Agriculture, Fisheries and Food (2023). Protocolo de Metafilaxia para el Complejo Respiratorio Bovino en Vacuno de Carne. https://www.mapa.gob.es/es/ganaderia/temas/sanidad-animal-higiene-ganadera/documentometafilaxiavacunocarne_tcm30-661702.pdf.

[21] Ministry of Agriculture, Fisheries and Food Informe de Base de Datos Técnico-Económica. Ejercicio Económico 2022. https://www.mapa.gob.es/es/ganaderia/temas/produccion-y-mercados-ganaderos/informebbdd_vacunoleche_2023_publicacion_web_tcm30-639443.pdf.

[22] Leruste H., Brscic M., Heutinck L.F., Visser E.K., Wolthuis-Fillerup M., Bokkers E.A., Stockhofe-Zurwieden N., Cozzi G., Gottardo F., Lensink B.J. (2012). The relationship between clinical signs of respiratory system disorders and lung lesions at slaughter in veal calves. Prev. Vet. Med..

[23] Macartney J.E., Bateman K.G., Ribble C.S. (2003). Health performance of feeder calves sold at conventional auctions versus special auctions of vaccinated or conditioned calves in Ontario. J. Am. Vet. Med. Assoc..

[24] Makoschey B., Berge A.C. (2021). Review on bovine respiratory syncytial virus and bovine parainfluenza—Usual suspects in bovine respiratory disease—A narrative review. BMC Vet. Res..

[25] Studer E., Schönecker L., Meylan M., Stucki D., Dijkman R., Holwerda M., Glaus A., Becker J. (2021). Prevalence of BRD-Related Viral Pathogens in the Upper Respiratory Tract of Swiss Veal Calves. Animals.

[26] Marzo E., Montbrau C., Moreno M.C., Roca M., Sitjà M., March R., Gow S., Lacoste S., Ellis J. (2021). NASYM, a live intranasal vaccine, protects young calves from bovine respiratory syncytial virus in the presence of maternal antibodies. Vet. Rec..

[27] Santo Tomás H., Barreto M., Vazquez B., Villoria P., Teixeira R., Sole M. (2023). Bovine Respiratory Disease Complex: Prevalence of the Main Respiratory Viruses Involved in Pneumonia in Spain. J. Anim. Sci. Res..

[28] Santo Tomás H., Teixeira R., Chacón G., Lázaro S., Sánchez-Matamorors A., Villoria P. (2023). Bovine Respiratory Disease Complex: Prevalence of the Different Bacteria Involved in Pneumonia in the Iberian Peninsula. J. Anim. Sci. Res..

[29] Peek S.F., Divers T.J. (2018). Rebhun’s Diseases of Dairy Cattle.

[30] Neethirajan S., Ragavan V., Weng X. (2018). Agro-defense: Biosensors for food from healthy crops and animals. Trends Food Sci. Technol..

[31] Hanzlicek G.A., White B.J., Mosier D., Renter D.G., Anderson D.E. (2010). Serial Evaluation of Physiologic, Pathological, and Behavioral Changes Related to Disease Progression of Experimentally Induced Mannheimia Haemolytica Pneumonia in Postweaned Calves. Am. J. Vet. Res..

[32] Kamel M.S., Davidson J.L., Verma M.S. (2024). Strategies for Bovine Respiratory Disease (BRD) Diagnosis and Prognosis: A Comprehensive Overview. Animals.

[33] (2019). Regulation (EU) 2019/6 of the European Parliament and of the Council of 11 December 2018 on veterinary medicinal products and repealing Directive 2001/82/EC. Off. J. Eur. Union.

[34] Gaudino M., Nagamine B., Ducatez M.F., Meyer G. (2022). Understanding the mechanisms of viral and bacterial coinfections in bovine respiratory disease: A comprehensive literature review of experimental evidence. Vet. Res..

[35] Zhang M., Hill J.E., Fernando C., Alexander T.W., Timsit E., van der Meer F., Huang Y. (2019). Respiratory Viruses Identified in Western Canadian Beef Cattle by Metagenomic Sequencing and Their Association with Bovine Respiratory Disease. Transbound. Emerg. Dis..

[36] Fulton R.W. (2020). Viruses in Bovine Respiratory Disease in North America: Knowledge Advances Using Genomic Testing. Vet. Clin. N. Am. Food Anim. Pract..

[37] Cuevas-Gómez I., McGee M., Sánchez J.M., O’Riordan E., Byrne N., McDaneld T., Earley B. (2021). Association between clinical respiratory signs, lung lesions detected by thoracic ultrasonography and growth performance in pre-weaned dairy calves. Ir. Vet. J..

[38] Cramer M.C., Ollivett T.L. (2019). Growth of preweaned, group-housed dairy calves diagnosed with respiratory disease using clinical respiratory scoring and thoracic ultrasound-A cohort study. J. Dairy Sci..

[39] Smith R.A. (1998). Impact of disease on feedlot performance: A review. J. Anim. Sci..

[40] Griffin D. (2014). The monster we don’t see: Subclinical BRD in beef cattle. Anim. Health Res. Rev..

[41] William P., Green L. (2007). Associations between Lung Lesions and Grade and Estimated Daily Live Weight Gain in Bull Beef at Slaughter. Cattle Pract..

[42] Buczinski S., Fecteau G., Dubuc J., Francoz D. (2018). Validation of a clinical scoring system for bovine respiratory disease complex diagnosis in preweaned dairy calves using a Bayesian framework. Prev. Vet. Med..

